# L-Carnitine Ameliorates Amiodarone-Mediated Alveolar Damage: Oxidative Stress Parameters, Inflammatory Markers, Histological and Ultrastructural Insights

**DOI:** 10.3390/ph17081004

**Published:** 2024-07-30

**Authors:** Samy A. Dawood, Ali Alsuheel Asseri, Ayed A. Shati, Refaat A. Eid, Basiouny El-Gamal, Mohamed Samir A. Zaki

**Affiliations:** 1 Department of Child Health, College of Medicine, King Khalid University, P.O. Box 62529, Abha 12573, Saudi Arabia; samyshorbagy8@hotmail.com (S.A.D.); alsoheel11@kku.edu.sa (A.A.A.); ashati@kku.edu.sa (A.A.S.); 2 Department of Pathology, College of Medicine, King Khalid University, P.O. Box 62529, Abha 12573, Saudi Arabia; 3 Clinical Biochemistry Department, College of Medicine, King Khalid University, P.O. Box 62529, Abha 12573, Saudi Arabia; basiouny_el_gamal@hotmail.com; 4 Department of Anatomy, College of Medicine, King Khalid University, P.O. Box 62529, Abha 12573, Saudi Arabia; mszaki@kku.edu.sa

**Keywords:** L-carnitine, amiodarone, histopathology, ultrastructure, oxidative markers, inflammatory markers

## Abstract

The aim of this study was to assess L-carnitine’s effects on adult male rats’ lung damage brought on by amiodarone, which is a potent antiarrhythmic with limited clinical efficacy due to potentially life-threatening amiodarone-induced lung damage. Because of the resemblance among the structural abnormalities in rats’ lungs that follows amiodarone medication and pulmonary toxicity in human beings, this animal model may be an appropriate example for this disease entity. Amiodarone produced pulmonary toxicity in twenty-four healthy male albino rats (150–180 g) over a period of 6 weeks. Four groups of six rats each were established: control, sham, amiodarone, and L-carnitine plus amiodarone. Histological, ultrastructural, oxidative stress, and inflammatory markers were determined during a 6-week exposure experiment. Amiodarone-induced lung damage in rats may be brought on due to oxidative stress producing significant pulmonary cytotoxicity, as evidenced by the disruption of the mitochondrial structure, severe fibrosis, and inflammatory response of the lung tissue. Lungs already exposed to such harmful effects may be partially protected by the antioxidant L-carnitine. Biochemical markers of lung damage brought on by amiodarone include lung tissue levels of the enzyme’s catalase, superoxide dismutase, and reduced glutathione. The levels of lipid peroxides in lung tissue measured as malondialdehyde increased significantly upon exposure to amiodarone. In addition, the levels of tumor necrosis factor alpha were significantly elevated in response to amiodarone. The effect of L-carnitine on amiodarone-induced pulmonary toxicity was studied in rats. It is interesting to note that the intake of L-carnitine in rats treated with amiodarone partially restored the biochemical and histopathological alterations brought on by amiodarone to their original levels. Tumor necrosis factor alpha levels were significantly reduced upon L-carnitine exposure. These results suggest that L-carnitine can be used to treat amiodarone-induced pulmonary dysfunction.

## 1. Introduction

In both humans and lower animals, the lungs serve as the primary respiratory organ. Due to exposure to hazardous substances, health hazards such fibrosis of the lungs, lung carcinoma, and pneumonia can affect the lung tissue. The hallmark of parenchymal lung involvement is thickening of the lung tissue, which results in gas exchange problems and, subsequently, hypoxia and hypercapnia. Pneumonocyte involvement and fibrous scarring both contribute to breathing issues [1,2].

Amiodarone (AMD) is one of the most useful antiarrhythmic drugs for the management of different types of arrhythmias [3]. Moreover, due to its serious side effects, especially pulmonary toxicity (inflammation and thickening of the alveolar septa, intra-alveolar inflammation, and pulmonary fibrosis), its use is limited [4,5]. High cumulative dosages of AMD have been demonstrated to have an impact on the lungs and extrapulmonary organs [6].

Several pulmonary toxicities have been associated with AMD, including acute respiratory distress syndrome, chronic interstitial pneumonitis, and organizing pneumonia [7], which can result in severe lung fibrosis [7,8]. Because of the existence of radial interstitial infiltrates, a continuous intake of AMD is frequently accompanied by harmful respiratory effects, such as breathing difficulties, wheeziness, and chest pain [9]. Older people are at a higher risk of developing pulmonary complications [10].

A principal factor in the development of AMD-induced pulmonary dysfunction is oxidative stress [5]. Oxidative stress, an imbalance between free radicals and the antioxidant defense system, is an important factor in the pathogenesis system that involves polyunsaturated membrane lipids. Oxidative stress normally is caused either due to an increased production of reactive oxygen species (ROS) or a decreased antioxidant capacity of cells, of which the former is the mechanism of amiodarone-induced cytotoxicity [11]. Since it is not possible to prevent the development of oxidative stress itself, it would be rational to increase the cellular levels of antioxidants, which is the hypothesis for evaluating the use of antioxidant agents to prevent amiodarone-induced cytotoxicity [12]. Consequently, the co-administration of antioxidants and amiodarone may increase the drug’s antiarrhythmic effects and promote its wider utilization [13]. 

Foods and nutrients shield the lungs and airways from oxidative damage, but a diet low in fruits and vegetables lowers antioxidant levels, making the body more vulnerable to inhaled irritants [14].

L-carnitine (LC) (γ-trimethylamino-β-hydroxybutyrate) is synthesized in vivo from methionine and lysine [15]. It is assumed that in normal circumstances, the biosynthesis of LC is sufficient to meet metabolic requirements. However, in several disease situations (apart from primary carnitine deficiency), oral LC supplements may be necessary as therapy [16]. The primary function of LC is to act as a carrier for the translocation of long-chain fatty acids from the cytosol into mitochondria for β-oxidation, hence sustaining the supply of energy [17]. However, besides this well-known effect, there is growing evidence that LC also plays a role in other physiological processes in humans and animals. Indeed, LC acts as a very potent ROS scavenger [18,19] and is known to have immunomodulatory properties in mammalian and avian species [20]. LC has been reported as a glucocorticoid mimicker because it activates the intracellular glucocorticoid receptor-a and modulates the expression of glucocorticoid-dependent genes during inflammation [21]. Glucocorticoids have a suppressive effect on the synthesis of proinflammatory cytokines by macrophages, and this effect is mimicked by LC [20].

### Malondialdehyde

The carnitine system comprises LC, acetyl carnitine, and cellular proteins for metabolism and transport to mitochondria for oxidation [22]. LC can be beneficial in maintaining metabolic flexibility via the optimization of mitochondrial function [23]. LC can inhibit the expression of inflammatory factors and oxidation to suppress the development of atherosclerosis by adjusting blood lipids in the myocardium of atherosclerotic Wistar rats [24].

This study’s objective was to investigate the underlying processes of rats’ lung injury from AMD and the probable contribution of LC in reducing respiratory hazards.

## 2. Results

### 2.1. Body Weight

Compared to control rats, AMD rats lost weight between the first week and the third week but restored it after this period. At the end of the experimental period, the body weight of different groups was significantly increased. On the other side, lung weight was significantly increased in AMD-induced rats compared to the control rats. Treatment of AMD rats with LC decreased the lung weight compared to AMD rats.

### 2.2. Histopathological

**Figure 1:**
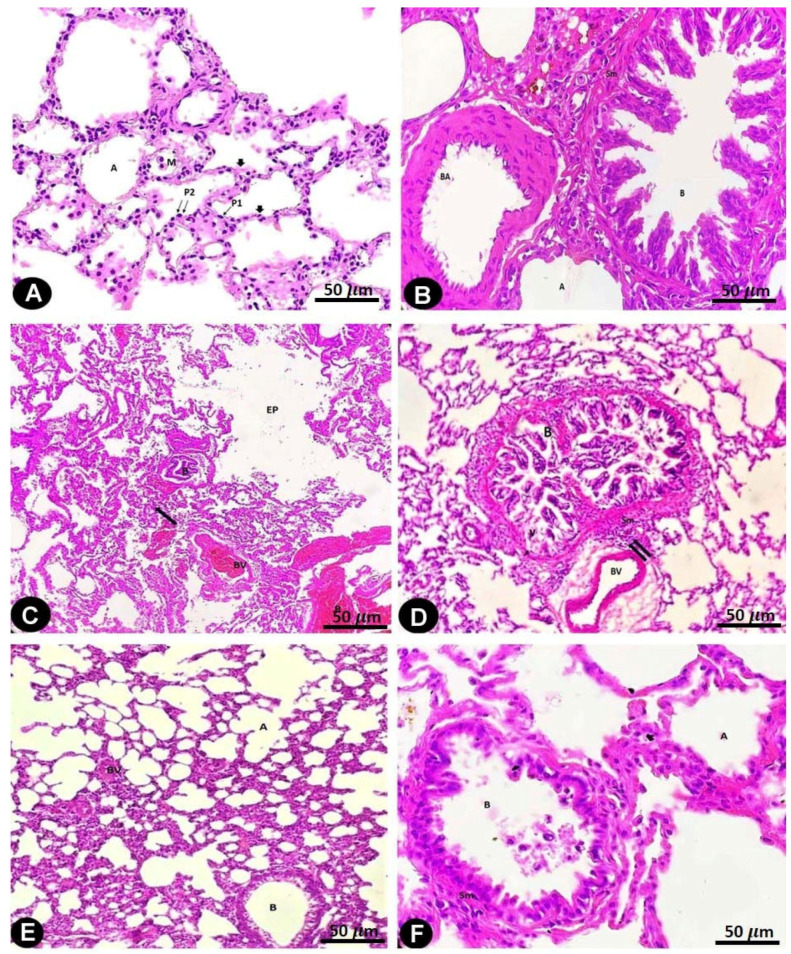
Hematoxylin- and eosin-stained sections of the rat lungs of all groups (bar = 50 µm).

In the H&E-stained specimens of the rat lungs of the control and sham-operated groups, the alveoli were bordered by type I pneumocyte cells, which possessed flat nuclei and an acidophilic cytoplasm; type II pneumocyte cells contained round nuclei and an acidophilic cytoplasm. It was established that smooth muscle cells surrounded the simple columnar epithelial cells that line the bronchioles in the lungs (Figure 1A,B and Table 1).

The lung architecture of the AMD-treated rats was altered, and mononuclear cell infiltration thickened the interalveolar septum. Extensive irregular emphysematous cavities developed due to damage to the interalveolar septum. Large numbers of inflammatory cells from mononuclear cells were observed to invade the interstitial tissues of the lungs. The blood vessels were found to be dilated and congested, and red blood cells were leaking from the vasculature. Bronchial involvement was observed in the form of peribranchial cell infiltration and epithelial cell desquamation (Figure 1C,D and Table 1).

The lung architecture of the LC- and AMD-treated rats, which comprised type I and type II pneumocyte-containing alveoli, was significantly improved after the rats received LC. Thin interalveolar septa surrounded and divided the alveoli, but only a small portion of these septa were thickened and contained mononuclear cell infiltrations. There were fewer areas of damaged interalveolar septa with large irregular emphysematous spaces. The bronchioles appeared to be lined with simple columnar epithelial cells that were encircled by smooth muscle cells. The walls of the bronchial arteries were thickened and showed slight peri bronchial cell infiltration (Figure 1E,F and Table 1).

Figure 1A: Rat lungs of the control and sham-operated groups displaying the typical lung structure of alveolar ducts, alveolar sacs, and alveoli (A), which are lined by an alveolar epithelium consisting of two types of cells: type I pneumocytes (P1) appear as flat cells with flat nuclei, and type II pneumocytes (P2) appear as rounded cells with rounded nuclei. The alveoli are separated from each other by thin interalveolar septa (arrow heads) consisting of the pneumocytes’ epithelial lining and loose connective tissue. A macrophage (M) can be seen in the wall of the alveoli. 

Figure 1B: Rat lungs of the control and sham-operated groups showing a bronchiole (B) encircled by a smooth muscle layer (Sm). Pulmonary arterioles (Art) and alveoli (A) can be seen. 

Figure 1C: Rat lung of the AMD-treated group showing collapsed bronchioles (B) having a thick smooth muscle layer (Sm). Also, dilated blood vessels (BV) that are congested with RBCs (R) can be observed. The collapse of the alveoli (A) with large irregular emphysematous air spaces (EP) and perivascular mononuclear cell infiltration (arrows) can be seen. 

Figure 1D: Rat lung of the AMD-treated group showing a bronchiole (B) lined with a disrupted epithelium showing an area of cytoplasmic vacuolation (v), dark pyknotic nuclei with an irregular arrangement (N) and thickening of the surrounding smooth muscle layer (Sm) with obliteration of its lumen by cellular debris (d). Peri bronchial cellular infiltration (arrows) and blood vessels (BV) with an irregular contour can be seen. 

Figure 1E: Rat lung of the LC plus AMD-treated group showing a bronchiole (B) with a normal smooth muscle layer thickness. The wall of the pulmonary arteriole (BV) has an apparent average thickness. Normal-sized air spaces, i.e., alveoli (A), can be seen. 

Figure 1F: Rat lung of the LC plus AMD-treated group showing a preserved bronchiole (B) with a normal smooth muscle layer (Sm) thickness. Normal-sized air spaces, i.e., alveoli (A), can be seen.

### 2.3. SEM

**Figure 2:**
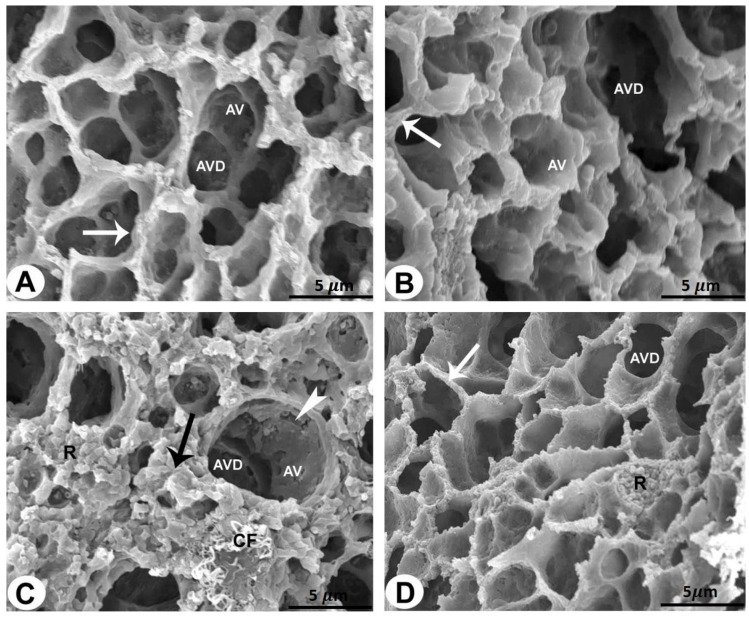
SEM of the rat lungs of all groups (bar = 5 µm). (**A**,**B**) Rat lungs of the control and sham-operated groups showing alveoli (AV) and alveolar ducts (AVD) surrounded by an enhanced alveolar ridge (arrows). (**C**) Rat lung of the AMD-treated group showing damaged and degenerated alveoli (AV) and alveolar ducts (AVD) of the lung sections. Destruction of alveolar wall lining (arrowheads) and increased amounts of erythrocytes (R) and collagen fibrils (CF) can be seen in the interalveolar septum (black arrow). (**D**) Rat lung of the LC plus AMD-treated group showing an improvement in the lung architecture. The alveoli (AV) and alveolar ducts (AVD) are surrounded by an enhanced alveolar ridge (arrows).

The rat lungs of the control and sham-operated groups showed multiple alveoli and air spaces with alveolar openings in their walls. A narrow interalveolar septum separated the adjacent alveoli (Figure 2A and Table 1).

The AMD-treated rat lung specimens clearly showed deterioration and damage to the alveoli and alveolar ducts. The interalveolar septum showed changes in the alveolar wall lining, an increased red blood cell count, and the deposition of collagen fibrils (Figure 2B,C and Table 1).

Examination of the lung specimens of the LC plus AMD-treated rats returned normal results with large alveoli. The alveolar openings to the air spaces and the thickness of the alveolar septum were of normal size (Figure 2D and Table 1).

### 2.4. TEM

**Figure 3:**
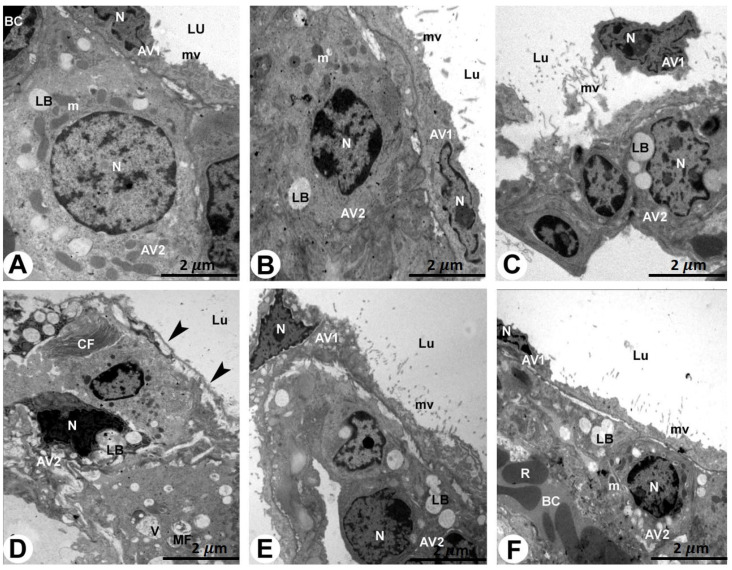
TEM of the rat lungs of all groups stained with uranyl acetate and lead citrate (bar = 2 µm).

The rat lungs of the control and sham-operated groups examined via electron microscopy showed a normal architecture, showing that the type I pneumocytes had mitochondria and flattened nuclei that were densely packed with microvilli. The cytoplasm of the type II pneumocytes was densely filled with mitochondria and lamellar structures and had a cuboidal shape with a rounded nucleus (Figure 3A,B and Table 1).

The specimens of the AMD-treated rat lungs showed obvious clinical changes such as dilated alveolar spaces separated by thickened alveolar septa, degraded pneumocytes, and extravasation of erythrocytes. The type I pneumocytes had distorted and irregular nuclei with unevenly distributed chromatin. The pyknotic nuclei and lamellar bodies of the type II pneumocytes were reduced, and microvilli were disrupted and lost. In addition, myelin figures and the deposition of collagen fibrils were observed. In addition, the type I and II pneumocytes had damaged mitochondria (Figure 3C–E and Table 1).

In the rat lungs of the LC plus AMD-treated group, the type I pneumocytes were normal, and the lungs’ structural integrity was retained. Round nuclei, lamellar bodies, numerous mitochondria, and projecting microvilli made the type II pneumocytes appear normal (Figure 3F and Table 1).

Figure 3A,B: Rat lungs of the control and sham-operated groups showing a normal lung architecture. 

 

A type I pneumocyte cell (AV1) with a fattened nucleus (N) and microvilli (mv) and a type II pneumocyte cell (AV2) with a rounded nucleus (N), lamellar bodies (LB), and mitochondria (m) can be seen. BC, blood capillary and Lu, lumen. 

 

Figure 3C–E: Rat lung of the AMD-treated group showing obvious apoptotic and degenerated lung sections. 

 

Figure 3C: A damaged and splitting type I pneumocyte cell (AV1) with destructed microvilli (mv) and irregularly shaped nucleus (N) of a type II pneumocyte cell (AV2). Lu, lumen.

 

Figure 3D,E: Absence of AV1 cells (arrowheads) lining the pneumocyte lumen (Lu). The apoptotic and degenerated AV2 cells show a pyknotic irregularly shaped nucleus (N) and destroyed and vacuolated lamellar bodies (LB). Collagen fibrils (CF) and myelin figures (MF) can also be seen. 

 

Figure 3F: Rat lung of the LC plus AMD-treated group showing an improvement in the lung architecture. 

A type I pneumocyte cell (AV1) with a fattened nucleus (N) and microvilli (mv) and a type II pneumocyte cell (AV2) with a rounded nucleus (N), lamellar bodies (LB), and mitochondria (m) can be seen. BC, blood capillary; R, red blood cells. Lu, lumen.

### 2.5. Biochemical Analysis

**Figure 4:**
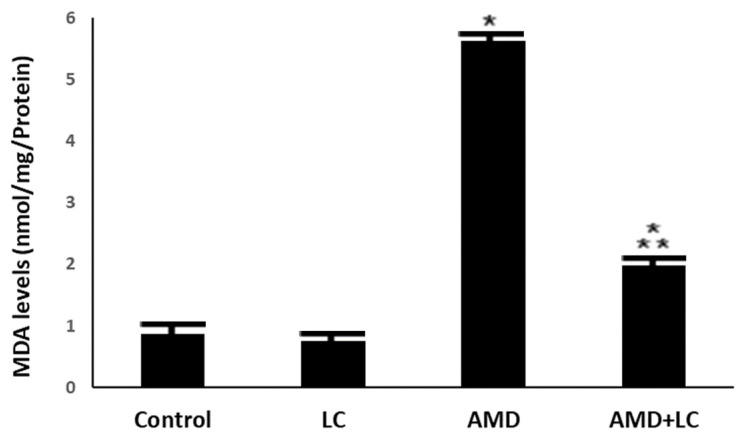
The levels of MDA in the studied groups. The increased MDA levels in the AMD group are significantly reduced when LC is added to the AMD group. One-way ANOVA followed by Tukey’s post hoc test was used for comparison between different groups. * *p* < 0.05 compared to the controls and 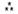 *p* < 0.05 in comparison to the AMD group.

The mean MDA levels increased significantly in the lung homogenates from the AMD-treated rats, which were statistically significant compared to the levels of the control and sham-operated groups (*p* < 0.001 and *p* < 0.0001, respectively). However, LC therapy in the LC- and AMD-treated group restored these parameters to normal values that were not statistically significant compared to the corresponding values of the control and sham-operated groups (*p* > 0.05) (Figure 4).

**Figure 5:**
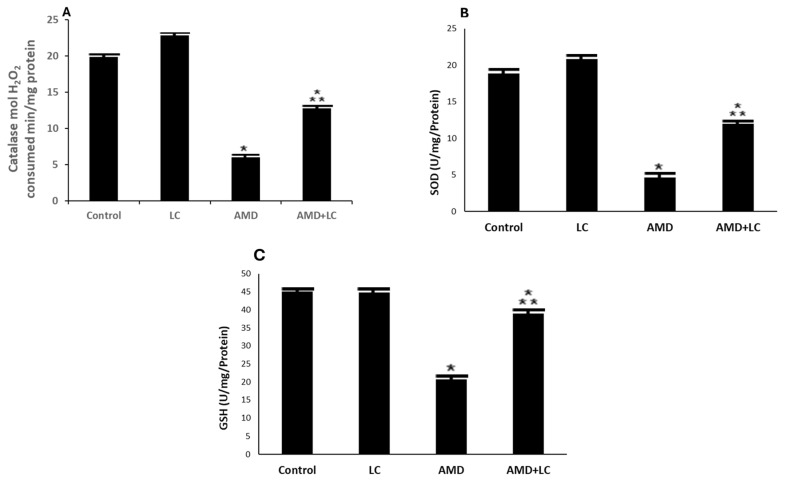
AMD group’s CAT (**A**), SOD (**B**), and GPX (**C**) activity was lower than that of the control group. The AMD group’s CAT and SOD activity significantly increased after LC administration. Data are represented as mean ± SD; *n* = 6. One-way ANOVA followed by Tukey’s post hoc test was used for comparison between different groups. * *p* < 0.05 compared to the controls; 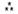 *p* < 0.05 in contrary to the AMD group. CAT: catalase; SOD: superoxide dismutase GPX: glutathione peroxidase.

The AMD-treated group showed a significant decrease in the CAT, SOD, and GSH levels in the lung homogenates, resulting in reductions in their CAT, SOD, and GSH values, which were statistically significant compared to the values of the control and sham-operated groups (Figure 5A–C).

In contrast, the LC- and AMD-treated group displayed a return to normal CAT, SOD, and GSH levels, and this was not statistically significant when compared to the results of the rat lungs of the control and sham-operated groups (*p* > 0.05).

**Figure 6:**
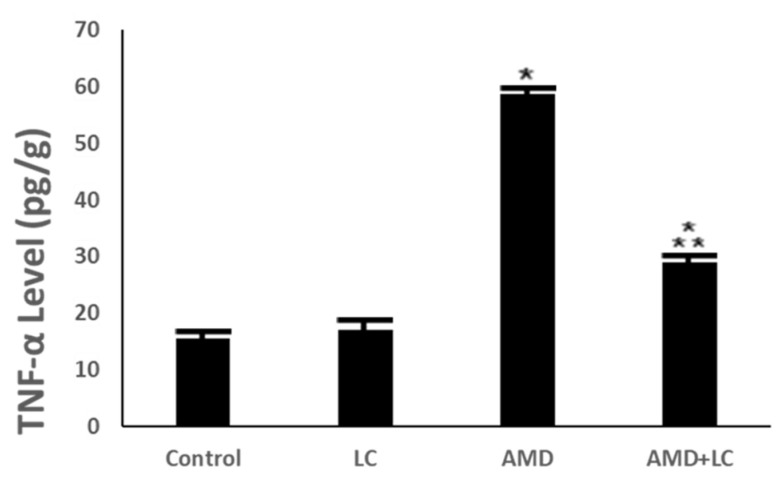
Levels of proinflammatory biomarker TNF-α in the studied groups. The AMD group’s TNF-α activity significantly reduced after LC administration. One-way ANOVA followed by Tukey’s post hoc test was used for comparison between different groups. * *p* < 0.05 compared to the controls and 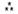 *p* < 0.05 in comparison to the AMD group. TNF-α: tumor necrosis factor-α.

The findings also revealed that, when compared to the controls, AMD significantly increased the TNF-α level. However, in rats receiving treatment with LC plus AMD exposure, there was a dramatic decrease in the TNF-α level (*p* < 0.001) (Figure 6).

## 3. Discussion

This study investigated AMD’s effects on the lungs, the effects of oxidative stress, and the effects of antioxidants to better understand how AMD causes lung damage in adult male albino rats. The current study sheds light on the protective potential of LC against AMD-induced pulmonary toxicity. This supports the clinical repositioning of drugs to lessen the side effects of AMD in AMD-receiving patients.

In the present study, AMD administration resulted in a significant reduction in the body weights of AMD rats in the first two weeks, increasing up to the end of the experimental period. This agrees with another study [25].

The histopathological results of this AMD study group revealed congestion of the pulmonary arteries and extravasated erythrocytes. These findings are consistent with the trends in previous research and might be caused by AMD’s direct harmful effects on the vessel wall [26]. In addition, damage to the interalveolar septum, the appearance of large emphysematous spaces, hyperinflation of the alveoli, and thickened alveolar septa were noticed in this group. Al-Shammari et al. [25] showed that histological abnormalities in the lungs of rats treated with AMD comprised interstitial capillary dilatation, granulomatous inflammation, interstitial pneumonia, and thickening of the alveolar wall. In addition, patchy fibrosis and cellular infiltration in the alveolar septum were previously noted [27].

Moreover, it was shown that in this group, rat lungs had a larger interalveolar collagen fiber area. There have been reports of areas of fibrosis in interstitial alveolar tissue [24] and increased collagen fibril deposition in the interstitium [6]. Long-term fibroblast proliferation, in accordance with a previous study [28], is the factor that triggers end-stage fibrosis.

However, AMD treatment additionally led to emphysematous spaces, regions of alveolar structure collapse, mononuclear cell infiltration, significant pneumocyte degeneration, and areas of consolidation. these findings are consistent with the findings of other researchers, who found that small bronchioles can become partially obstructed due to the detachment of epithelial cells and infiltrations of inflammatory cells [29].

The histopathological alterations in AMD-induced lungs in this study were confirmed ultra-structurally. The pneumocytes type I and II of the AMD-treated rat lungs had damaged mitochondria. Oxidative stress may contribute to the development of lung damage induced by AMD [30]. Studies have linked changes in AMD to toxicity, hypersensitivity, increased oxidation indicators, free radical production [31], phospholipidosis activation, mitochondrial malfunction, and inflammatory mediator release [23,32]. Mitochondria adapt to environmental stresses through an adaptive stress response, which is responsible for oxidative-phosphorylation-induced ROS production, fatty acid oxidation, and calcium flux regulation [33]. LC’s antioxidant properties counteract age-related mitochondrial dysfunction in rats [22]. A common histopathologic finding of AMD-induced lung injury is diffuse alveolar damage. This damage can be split into two stages: an acute exudative phase and late repair phase. Alveolar and interstitial edemas, as well as hyaline membranes, are characteristics of the exudative phase, which begins within the first week of lung damage [22]. Type II pneumocyte proliferation and interstitial fibrosis characterize the repair phase, which follows the exudative phase by 1–2 weeks [22].

Disrupted microvilli, distorted and irregular nuclei with unevenly distributed chromatin, lamellar bodies, and the deposition of collagen fibrils were also evident in relation to AMD- treated lungs in this current investigation. Amiodarone accumulates in pneumocytes associated with hyperplasia and septal thickening [34]. Foamy alveolar macrophages and cytoplasmic lamellar bodies containing surfactant-like material are typical of amiodarone exposure and do not necessarily indicate clinically significant toxicity [35].

According to the biochemical analysis conducted in this experiment, AMD administration generated a significant elevation in lung MDA content. Malondialdehyde (MDA) is a byproduct of the peroxidation of polyunsaturated fatty acids that occurs within various cell types. Oxidative stress markers indicate MDA overproduction, causing membrane lipid peroxidation and endothelium damage [36]. A previous study showed that the pathogenesis of lung damage brought on by AMD heavily relies on oxidative stress [37].

Furthermore, the biochemical examination conducted in this study revealed that the CAT and GSH levels of the AMD-treated group significantly decreased. To prevent oxidative damage, these antioxidant enzymes form the first line of defense [38]. One of the enzymatic antioxidant defense mechanisms that removes free radicals is CAT [39]. Rats treated with AMD had significantly lower CAT scores, suggesting that AMD-induced lung damage may be caused by oxidative stress [40]. The primary antioxidant in the lining of the respiratory system is GSH, a thiol tripeptide comprising glutamate, cysteine, and glycine [40]. Reduced bronchoalveolar lavage decreases the creation of ROS products such as hydroxyl, H_2_O_2_, hypochlorous acid, and lipid peroxyl radicals after the intake of oxidants like allergens, ozone, and cigarette smoke. Due to GSH’s evasive ability, GSH levels have been identified as a hallmark of various lung diseases [41]. GSH is an important molecule that wards off oxidative damage and keeps cells in a reducing medium [42].

In the current investigation’s biochemical examination, the TNF-α levels in the AMD-treated group significantly increased. Tumor necrosis factor-α caused the overproduction of nitric oxide in the liver, which reacts with superoxide radical, forming peroxynitrite, a potent oxidizing agent. Peroxynitrite can react directly with sulfahydryl residues in cell membranes as well as with DNA, leading to lipid peroxidation and cytotoxicity [43].

Levels of tumor necrosis factor alpha (TNF-α) have been linked to inflammatory respiratory disorders, which include asthma, chronic obstructive pulmonary disease, and sarcoidosis. TNF-α influences disease pathophysiology by enhancing tissue remodeling and inflammatory mediator production. TNF-α-targeting biologics may potentially be a very potent treatment approach [44]. T-helper cell imbalance and excess cytokine production are associated with AMD-induced pulmonary toxicity and have been shown to result in significant increases in circulating TNF-α levels [45]. Significant amounts of TNF-α were released from alveolar macrophages isolated from AMD-treated rats [46].

We found that the administration of LC improved the histological and biochemical changes in lung tissue caused by AMD. LC maintains mitochondrial function by reducing ROS and endoplasmic reticulum stress during autophagy-induced cell death [47]. It has been proposed that LC contributes to ATP production by removing fatty acids from mitochondria during oxidation, resulting in a significant reduction in oxygen and ROS concentrations during ATP creation. Due to its capacity to boost the activity of antioxidant enzymes, LC is thought to have antioxidant properties [48,49]. Another form of protection provided by LC is the increased antioxidant capacity of cellular mitochondria [50].

In this study, LC significantly increased CAT, SOD, and GSH levels. This is in line with the past studies that investigated the anti-inflammatory and free-radical-scavenging abilities of LC [51]. Supplementation with LC was associated with decreased TNF-α and MDA levels and increased SOD levels [52]. The results of this study agree with those of ref. [52], who demonstrated a significant increase in antioxidant enzyme levels with LC. In this study, TNF-α was markedly decreased in the LC- and AMD-treated group compared to the AMD-treated group. Following LC therapy, there was a considerable reduction in lung TNF-α levels [52].

## 4. Materials and Methods

### 4.1. Animals

In total, 24 Wistar albino rats weighing 150–200 gm were provided with unlimited access to food and water as they acclimated to the lab environment for two weeks. The experimental protocols were designed using the European Community Directive (86/609/EEC), and the national rules on animal care and all experiments were carried out in accordance with the NIH Guidelines for the Care and Use of Laboratory Animals 8th edition. The experimental protocol used in this study was approved by the Animal Ethics Committee (ECM#2024-505) of the Faculty of Medicine, King Khalid University, Saudia.

### 4.2. Chemicals Yes Revised

L-carnitine (LC) (3-hydroxy, 4-trimethylamino butyric acid) and amiodarone (AMD) (C25 H29 I2 NO3) were donated by Sigma Chemical Company (St. Louis, MO, USA). Hematoxylin and eosin (H&E) were purchased from Abcam (Boston, MA, USA). Malondialdehyde (MDA) (cat. MBS268427), catalase (CAT) (cat MBS9712526), superoxide dismutase (SOD) (cat MBS729914), reduced glutathione (GSH) (cat MBS046356), and tumor necrosis factor-α (TNF-α) (cat MBS175908) were analyzed using special enzyme-linked immunosorbent assay (ELISA) kits for rats, My BioSource, (San Diego, CA, USA).

### 4.3. Experimental Approach

A total of four groups of six animals each were established. The rats in Group I (control) were orally administered 0.5 cc of distilled water for 30 days. The Group II (sham) rats received 100 mg/kg of LC orally for 30 days. The Group III (AMD-treated) rats were orally administered 30 mg/kg body weight (BW) of AMD daily for sixty days [38]. The Group IV (LC- and AMD-treated) rats were orally administered LC (100 mg/kg BW) for 30 days after 60 days of exposure to AMD orally (30 mg/kg BW) [40].

### 4.4. Samples Collection

At the end of experimental period, rats of all groups were weighed and were anaesthetized using ether. Their lungs were dissociated carefully and fixed subsequently in 10% neutral formaldehyde for histopathological assessments. One-half of the lungs was homogenized in chilly phosphate-buffered saline (PBS, pH 7.4). The prepared homogenate was centrifuged (18,000× *g*, 4 °C) for 30 min.

### 4.5. Light Microscopic Examination

Small portions of each rat lung were promptly cut up and placed in a 10% formalin-saline fixative for 24 h. The samples were then cleaned and dehydrated in ethanol concentrations of 70%, 90%, and 100%. They spent two hours being cleared of xylene. Soft paraffin wax was impregnated and embedded for three hours at 45 to 50 °C, while hard paraffin wax was embedded for one hour at 60 °C. On 5 µm sections of paraffin blocks, hematoxylin and eosin (H&E) staining was carried out [53]. Using an Olympus BX50 light microscope (Tokyo, Japan), histopathological analysis was performed. The King Khalid University, College of Medicine’s Department of Pathology examined the samples.

### 4.6. Scanning Electron Microscopy (SEM) Examination

After being immersed in 2.5% glutaraldehyde for 12 h, lung specimens were rinsed with 0.1 M phosphate buffer and then incubated for 1 h at 4 °C in a solution of 0.1 M osmium tetroxide in 1% phosphate-buffered solution. The lung samples were then cleaned in three baths of double-distilled water for 23 min each before being placed in a bath of 1% aqueous tannic acid for 1 h at 4 °C. The specimens were then dehydrated for ten minutes each in ascending grades of ethanol: 50%, 70%, 90%, and 95%. Then, an SPI apparatus, namely a liquid CO_2_ critical point dryer, was used to dry the samples. Using an SPI Module Vac/Sputter, the samples were coated with gold and mounted on an aluminum stub [54]. SEM (JEOL JSM–6390LV—Tokyo, Japan) was used for analysis at the King Khalid University College of Medicine’s Pathology Department. This approach can enable rapid image classification and microstructural feature mapping needed for emerging high-throughput characterization and autonomous microscope platforms [55].

### 4.7. Transmission Electron Microscopy (TEM) Examination

For the TEM examination, 0.1 M phosphate buffer (pH 7.4) and 2.5% glutaraldehyde were used to fix fresh lung sections into one mm^3^ pieces, which were subsequently post-fixed with 1% osmium tetroxide. Then, the specimens were embedded in spur’s resin and placed in an oven at 60 °C. Uranyl acetate and lead citrate were used as counterstains on ultrathin sections placed on copper grids [56]. A JEM-1011 (JEOL, Tokyo, Japan) TEM was used for analysis at the King Khalid University College of Medicine’s Pathology Department. Understanding the subcellular elements of cells and tissues first requires quantifying the organelles in TEM images. The National Institutes of Health originally developed an open-source program that may be used to measure organelles in TEM images (https://imagej.nih.gov/ij/plugins/quadrant-picking/index.html, accessed on 24 July 2024). The program may be used to examine the mitochondrial/endoplasmic reticulum interaction and measure parameters including mitochondrial length, width, area, and circularity [57].

### 4.8. Biochemical Analysis

#### 4.8.1. Preparation of Lung Homogenate

Ten percent (*w*/*v*) lung homogenates were created by homogenizing lung samples in five mmol/L of Tris-HCl buffer plus two mmol/L ethylene diamine tetra-acetic acid (EDTA), pH 7.4. The homogenates were centrifuged for 10 min at 4 °C and 1000 rpm to separate the supernatants, which were then immediately used to analyze the oxidant–antioxidant status.

#### 4.8.2. Antioxidant Enzyme Markers and Malondialdehyde (MDA)

Antioxidant enzyme activity, such as that of CAT [58], SOD [59], and (GSH) [60], and the MDA level which is a non-enzymatic marker of oxidative stress specifically for lipid peroxidation [61], were determined in rat lung homogenates utilizing the instruction manual for commercially accessible kits. ELISA kits specifically designed for rat analyses were utilized to evaluate antioxidant enzyme assays used in this study. The kits were sourced from My BioSource in California (USA).

#### 4.8.3. Estimation of TNF-α Marker

The level of the inflammatory cytokine TNF-α in the lung homogenates was measured using Rat Inflammation Array Q1 (Ray Biotech, QAR-INF-1, Peachtree Corners, GA, USA) according to the manufacturer’s instructions. The measurements were performed at 450 nm using a microplate-reading spectrophotometer (Thermo Scientific Multiskan FC, Waltham, MA, USA). The levels of TNF-α are expressed as pg/mg of protein [62].

### 4.9. Statistical Analysis

Data were analyzed using the SPSS Software program version 29 (IBM SPSS Statistics for Windows, Armonk, NY, USA: IBM Corp). The Shapiro test was used to assess the normality of the data. Continuous variables are presented as the mean ± SD (standard deviation). The one-way ANOVA test was used to compare the means, followed by Tukey’s post hoc test. The results were considered nonsignificant when the probability of error was more than 5% (*p* > 0.05) and significant when the probability of error was less than 5% (*p* ≤ 0.05) [63].

## 5. Conclusions

These results indicate that LC considerably reduces the histological and functional damage of the lung that is associated with AMD treatment. As AMD is believed to induce oxidative stress—which initiates a whole chain of complex biochemical reactions resulting in cell and nuclear damages by free radicals—we showed here that when concomitantly administered, LC acts partially to protect and repair the structural and functional damage caused to lung alveoli by engaging the existent antioxidative potential at the level of pulmonary tissues. Thus, the co-administration of antioxidants with amiodarone may lead to a more widespread application of amiodarone.

## Figures and Tables

**Table 1:** The percentage scoring system of the LC and AMD histopathological and ultrastructural features of all groups of rats.

Lung Structures		Percentages			
		Control Group	LC Group	AMD Group	LC Plus AMD Group
Histopathological Analysis	Alveolar duct	0–1%	1–2%	55–58%	1–4%
	Alveolar sacs	0–1%	2–3%	35–40%	2–3%
	Alveoli	0–1%	0–2%	29–37%	1–2%
	Alveolar epithelium	0–1%	0–1%	25–28%	2–3%
	Type I pneumocytes	0–1%	1–2%	35–41%	2–3%
	Type II pneumocytes	0–1%	1–2%	25–28%	1–4%
	Interalveolar septum	0–1%	1–2%	34–45%	1–2%
	Bronchiole	0–1%	0–2%	65–78%	1–3%
Ultrastructural Analysis	SEM Analysis				
	● Alveolar duct	0–1%	1–2%	52–54%	1–7%
	● Alveoli	0–1%	1–2%	24–30%	1–5%
	● Alveolar enhance ridge	0–1%	1–2%	35–40%	2–3%
	● Collagen fibrils	0–1%	1–2%	14–28%	1–2%
	● Erythrocytes	0–1%	1–2%	12–21%	1–3%
	TEM Analysis				
	● Type I pneumocytes	0–1%	1–2%	37–43%	1–2%
	● Type II pneumocytes	0–1%	1–2%	26–31%	1–4%
	● Blood capillary	0–1%	1–2%	36–44%	2–3%
	● Collagen fibrils	0–1%	1–2%	16–29%	1–2%
	● Myelin figures	0–1%	1–2%	8–11%	0–1%

## Data Availability

Upon reasonable request, the corresponding author will provide the datasets used and/or analyzed in this work.
